# Consensus statement on standards and guidelines for the molecular diagnostics of Alport syndrome: refining the ACMG criteria

**DOI:** 10.1038/s41431-021-00858-1

**Published:** 2021-04-15

**Authors:** Judy Savige, Helen Storey, Elizabeth Watson, Jens Michael Hertz, Constantinos Deltas, Alessandra Renieri, Francesca Mari, Pascale Hilbert, Pavlina Plevova, Peter Byers, Agne Cerkauskaite, Martin Gregory, Rimante Cerkauskiene, Danica Galesic Ljubanovic, Francesca Becherucci, Carmela Errichiello, Laura Massella, Valeria Aiello, Rachel Lennon, Louise Hopkinson, Ania Koziell, Adrian Lungu, Hansjorg Martin Rothe, Julia Hoefele, Miriam Zacchia, Tamara Nikuseva Martic, Asheeta Gupta, Albertien van Eerde, Susie Gear, Samuela Landini, Viviana Palazzo, Laith al-Rabadi, Kathleen Claes, Anniek Corveleyn, Evelien Van Hoof, Micheel van Geel, Maggie Williams, Emma Ashton, Hendica Belge, Elisabeth Ars, Agnieszka Bierzynska, Concetta Gangemi, Beata S. Lipska-Ziętkiewicz

**Affiliations:** 1 Department of Medicine (MH and NH), The University of Melbourne, Parkville, VIC Australia; 2 Molecular Genetics, Viapath Laboratories, Guy’s Hospital, London, UK; 3 Elizabeth Watson, South West Genomic Laboratory Hub, North Bristol Trust, Bristol, UK; 4 Jens Michael Hertz, Department of Clinical Genetics, Odense University Hospital, Odense, Denmark; 5 Center of Excellence in Biobanking and Biomedical Research and Molecule Medicine Center, University of Cyprus, Nicosia, Cyprus; 6 Medical Genetics, University of Siena, Siena, Italy; 7 Institute de Pathologie et de Genetique ASBL, Departement de Biologie Moleculaire, Gosselies, Belgium; 8 Department of Medical Genetics, and Department of Biomedical Sciences, University Hospital of Ostrava, Ostrava, Czech Republic; 9 Departments of Pathology and Medicine (Medical Genetics), University of Washington, Seattle, WA USA; 10 Institute of Biomedical Sciences, Faculty of Medicine, Vilnius University, Vilnius, Lithuania; 11 Division of Nephrology, Department of Medicine, University of Utah Health, Salt Lake City, UT USA; 12 Clinic of Pediatrics, Institute of Clinical Medicine, Faculty of Medicine, Vilnius University, Vilnius, Lithuania; 13 Department of Pathology, University of Zagreb, School of Medicine, Dubrava University Hospital, Zagreb, Croatia; 14 Nephrology Unit and Meyer Children’s University Hospital, Firenze, Italy; 15 Division of Nephrology and Dialysis, Bambino Gesù Children’s Hospital, IRCCS, Rome, Italy; 16 Department of Experimental Diagnostic and Specialty Medicine (DIMES), Nephrology, Dialysis and Renal Transplant Unit, S. Orsola Hospital, University of Bologna, Bologna, Italy; 17 Wellcome Centre for Cell-Matrix Research, Division of Cell-Matrix Biology and Regenerative Medicine, School of Biological Sciences, Faculty of Biology Medicine and Health, The University of Manchester, Manchester, UK; 18 School of Immunology and Microbial Sciences, Faculty of Life Sciences, King’s College London, London, UK; 19 Fundeni Clinical Institute, Pediatric Nephrology Department, Bucharest, Romania; 20 Centre for Nephrology and Metabolic Disorders, Weisswasser, Germany; 21 Institute of Human Genetics, Technical University of Munich, München, Germany; 22 Nephrology Unit, University of Campania, Naples, Italy; 23 Department of Biology, School of Medicine University of Zagreb, Zagreb, Croatia; 24 Birmingham Children’s Hospital, Birmingham, UK; 25 Departments of Genetics and Center for Molecular Medicine, University Medical Center, Utrecht University, Utrecht, The Netherlands; 26 Alport UK, Gloucester, UK; 27 Medical Genetics Unit, Department of Clinical and Experimental Biomedical Sciences “Mario Serio”, University of Florence, Florence, Italy; 28 Medical Genetics Unit, Meyer Children’s University Hospital, Florence, Italy; 29 Health Sciences Centre, University of UTAH, Salt Lake City, UT USA; 30 Department of Nephrology and Renal Transplantation, University Hospitals Leuven, Leuven, Belgium; 31 Center for Human Genetics, University Hospitals and KU Leuven, Leuven, Belgium; 32 Department of Clinical Genetics, Maastricht University Medical Center, Maastricht, The Netherlands; 33 Bristol Genetics Laboratory Pathology Sciences, Southmead Hospital, Bristol, UK; 34 North East Thames Regional Genetics Laboratory, Great Ormond Street Hospital, London, UK; 35 Department of Physiology, Radboud Institute for Molecular Life Sciences, Radboud University Medical Center, Nijmegen, The Netherlands; 36 Inherited Kidney Disorders, Fundacio Puigvert, Universitat Autonoma de Barcelona, Barcelona, Spain; 37 Bristol Renal Unit, Bristol Medical School, University of Bristol, Bristol, UK; 38 Division of Nephrology and Dialysis, University Hospital of Verona, Verona, Italy; 39 Centre for Rare Diseases, and Clinical Genetics Unit, Medical University of Gdansk, Gdansk, Poland

**Keywords:** Diseases, Alport syndrome

## Abstract

The recent Chandos House meeting of the Alport Variant Collaborative extended the indications for screening for pathogenic variants in the *COL4A5, COL4A3* and *COL4A4* genes beyond the classical Alport phenotype (haematuria, renal failure; family history of haematuria or renal failure) to include persistent proteinuria, steroid-resistant nephrotic syndrome, focal and segmental glomerulosclerosis (FSGS), familial IgA glomerulonephritis and end-stage kidney failure without an obvious cause. The meeting refined the ACMG criteria for variant assessment for the Alport genes (*COL4A3–5)*. It identified ‘mutational hotspots’ (PM1) in the collagen IV α5, α3 and α4 chains including position 1 Glycine residues in the Gly-X-Y repeats in the intermediate collagenous domains; and Cysteine residues in the carboxy non-collagenous domain (PP3). It considered that ‘well-established’ functional assays (PS3, BS3) were still mainly research tools but sequencing and minigene assays were commonly used to confirm splicing variants. It was not possible to define the Minor Allele Frequency (MAF) threshold above which variants were considered Benign (BA1, BS1), because of the different modes of inheritances of Alport syndrome, and the occurrence of hypomorphic variants (often Glycine adjacent to a non-collagenous interruption) and local founder effects. Heterozygous *COL4A3* and *COL4A4* variants were common ‘incidental’ findings also present in normal reference databases. The recognition and interpretation of hypomorphic variants in the *COL4A3–COL4A5* genes remains a challenge.

## Introduction

Estimates of the prevalence of Alport syndrome vary from one in 5,000 to one in 53,000 of the population [1, 2], but the frequent finding of likely pathogenic *COL4A5* variants in normal reference datasets and cohorts with renal failure suggests that X-linked disease affects closer to closer to one in 5,000 [3, 4]. This is consistent with Alport syndrome being the second commonest cause of inherited kidney failure after autosomal dominant Polycystic Kidney Disease. It also means that many variants are undetected and that even apparently normal individuals have pathogenic variants in the *COL4A3–COL4A5* genes.

Inheritance of Alport syndrome is X-linked (OMIM301050), autosomal recessive (OMIM 203780), digenic (typically with a pathogenic variant in each of *COL4A3* and *COL4A4* [5]) or autosomal dominant (OMIM 104200, or thin basement membrane nephropathy, due to heterozygous *COL4A3* or *COL4A4* variants [6]). The typical clinical features of Alport syndrome are persistent microscopic haematuria, end-stage kidney failure, and often a family history of haematuria or renal failure [7]. Hearing loss and non-nephrotic range proteinuria are common. Ocular abnormalities such as lenticonus with abnormal lens protrusion, or fleck retinopathy may be present [7]. Clinical features overlap between boys and men with X-linked disease, and in boys and girls, men and women with recessive or digenic inheritance [5, 8]. Many women with X-linked Alport syndrome, and males and females with heterozygous *COL4A3* or *COL4A4* variants, or sometimes digenic *COL4A3* and *COL4A4* variants, have only microscopic haematuria [5, 8, 9].

In February 2020, a group of 47 medical and scientific specialists from three continents, all with an interest in the molecular diagnostics Alport syndrome (the ‘Alport Variant Collaborative’), met in London to review the current recommendations for genetic testing for Alport syndrome and to further refine the American College of Medical Genetics/Association of Molecular Pathologists (ACMG/AMP) criteria for evaluating variants in the *COL4A3–COL4A5* genes that have been published previously [8, 10]. The group comprised adult (*n* = 12) and paediatrics (*n* = 6) nephrologists, histopathologists (*n* = 2), geneticists (*n* = 17), laboratory scientists (*n* = 4), researchers (*n* = 2), industry scientists (*n* = 3) and a representative of the Alport UK Patient Support group (*n* = 1). Attendees had submitted difficult-to-evaluate *COL4A3–COL4A5* variants, which were precirculated, and they spent two sessions at the meeting examining these variants, and discussing the evidence for pathogenicity. Their conclusions have been summarised into the following guidelines for genetic testing for Alport syndrome for use by molecular testing laboratories. A draft of these guidelines was then sent to all attendees, modified based on their comments, and the final document approved by the group prior to submission. Decisions about individual variants are ongoing with further clinical data being sought in order to reach at least two-thirds consensus on the assertions.

## Broadening the phenotype

Increasingly, pathogenic variants in *COL4A3–COL4A5* are found in individuals with proteinuria (‘nephrotic syndrome’, ‘nephrotic range proteinuria’, ‘steroid-resistant nephrotic syndrome’) or with the renal biopsy finding of focal and segmental glomerulosclerosis (FSGS)[11, 12]. FSGS is one of the commonest causes of glomerular disease resulting in progressive renal failure, and pathogenic *COL4A3–COL4A5* variants are the commonest cause of adult-onset FSGS ranging from 5% to 20% of cases with sporadic or familial disease respectively [11, 12]. In such cases the glomerular basement membrane does not necessarily have the lamellation typical of Alport syndrome and *COL4A3–COL4A5* variants may be associated with an older age at end-stage kidney failure [13]. Pathogenic variants in any of the *COL4A3*–*COL4A5* genes are also found in up to 10% of individuals with renal failure where the cause was not known [3]. In addition families with IgA glomerulonephritis not uncommonly have mutations in the *COL4A3–COL4A5* genes [14]. Finally there are reports of *COL4A3–COL4A5* variants being associated with renal cysts where autosomal dominant polycystic kidney disease has been excluded [15, 16].

Thus, not only individuals with the typical features of Alport syndrome should undergo genetic testing, but also those with proteinuria or FSGS, with familial IgA glomerulonephritis or with end-stage kidney failure where there is no obvious cause (Table 1). For individuals with proteinuria or FSGS, or end-stage kidney failure, the presence of haematuria or a family history of haematuria, and in the case of IgA disease, a family history of haematuria, all make the detection of a pathogenic *COL4A3–COL4A5* variant more likely. However the meeting considered there was currently insufficient evidence to recommend that individuals with cystic kidney disease should also be tested for *COL4A3–COL4A5* variants.

**Table 1:** Recommendations for genetic testing for Alport syndrome.

Clinical spectrum	The typical clinical features of Alport syndrome are persistent haematuria, sometimes with progressive renal failure, together with a family history of haematuria or renal failure. Proteinuria, a hearing loss or a lamellated or thinned glomerular basement membrane may be present. Other phenotypes associated with pathogenic variants in the *COL4A3–COL4A5* genes and hence Alport syndrome include persistent proteinuria, steroid-resistant nephrotic syndrome and focal and segmental glomerulosclerosis (FSGS); familial IgA glomerulonephritis; and end-stage kidney failure of unknown cause. Pathogenic *COL4A3–COL4A5* variants are the commonest genetic abnormality found in all these phenotypes.
Genetic testing	The most accurate test for the detection of a causative pathogenic variant in the *COL4A3–COL4A5* genes is comprehensive parallel genetic testing of the entire coding sequences of all three *COL4A5*, *COL4A3* and *COL4A4* genes. Where Alport syndrome is suspected, genetic testing for diagnosis should take precedence over even a renal biopsy. Where a causative variant is demonstrated, a renal biopsy may not be necessary. The *COL4A3–COL4A5* genes should also be included in multigene Massively Parallel Sequencing renal disease panels for the extended Alport phenotypes. The Alport syndrome-specific modifications of the ACMG standards and guidelines for the Interpretation of Sequence Variants apply.

## Genetic testing of the *COL4A3–COL4A5* genes

Finding a *COL4A3–COL4A5* variant that affects the structure or function of a collagen IV α chain confirms the diagnosis of Alport syndrome, and indicates that other family members should be investigated. Genetic testing also identifies the mode of inheritance, and which family members who are at risk of being affected. End-stage kidney failure is very likely in hemizygous *COL4A5* males (90% by the age of 40 years) and individuals with biallelic defects in *COL4A*3 or *COL4A4*, but much less common in females with a heterozygous *COL4A5* variant (15–30% by 60 years) and a person with a heterozygous pathogenic variant in *COL4A3 or COL4A4* [17]. The nature of individual variants also indicates the likelihood of early-onset kidney failure and extra-renal features such as ocular abnormalities [18, 19]. Hearing loss is common with X-linked and recessive disease [19]. Targeted diagnostics can be used to identify other affected family members, to ensure that an affected person does not act as a kidney donor, and in preimplantation genetic diagnosis [8]. Where a variant is identified in a person referred with FSGS for testing, the finding of a causative pathogenic variant in one of the *COL4A3–COL4A5* genes means that treatment with corticosteroids or immunosuppressants is not usually useful [4, 20, 21], and that this type of FSGS does not typically recur after renal transplantation.

MPS usually identifies at least 80% of disease-causing variants in *COL4A3–COL4A5* where Alport syndrome is suspected clinically [22] because of microscopic haematuria or progressive kidney failure, together with a positive family history of haematuria or renal failure. Where a causative variant or variants are not found, it is important to ensure that the genes are sufficiently ‘covered’ by the sequencing technique. Other explanations for the inability to detect pathogenic variants are the large genomic imbalances encompassing one or more exons (such as deletions, duplications or inversions) that MPS detects less well; a location deep within an intron or regulatory element or affecting a non-canonical splice site (for example, 5 nucleotides from the intron-exon boundary); there being insufficient evidence for pathogenicity and the variant being assessed as a Variant of Uncertain Significance (VUS); the variant affecting a processing enzyme or chaperone rather than a *COL4* gene, as occurs in about 10% of cases of osteogenesis imperfecta; [23] and rarely because of mosaicism. Sometimes the disease is simply not Alport syndrome. Phenocopies of Alport syndrome where there is predominantly haematuria include Fechtner syndrome (OMIM155100), fibronectin glomerulopathy (OMIM 601894), Nail-Patella syndrome (OMIM 161200), Hereditary Angiopathy, Nephropathy and muscle Cramps (OMIM 611773), Dense deposit disease (OMIM 134370) and *CFHR5* deficiency (OMIM 614809). However increasingly pathogenic variants are found in genes affected in proteinuric diseases.

More pathogenic variants are known for *COL4A5* than for *COL4A3* and *COL4A4*, and these are missense in 60%, nonsense in 10%, canonical splice sites in 10% and frameshifts in 20% (databases.lovd.nl/shared/genes/*COL4A5*). About half the pathogenic *COL4A3–COL4A5* variants found in diagnostic laboartories are novel. There appear to be some changes in the types of pathogenic variants found in the last years. Fewer large deletions have been reported possibly because MPS detects these less well, or because the more severe disease that these people have means that they have already been studied. In addition, more non-canonical splice site variants, and non-Gly substitutions in the collagenous and carboxy NC1 domains have been reported.

## Definition of pathogenicity

The definition of pathogenicity has become less clear for sequence variants in *COL4A3–COL4A5*. Previous meetings have concluded that the demonstration of a pathogenic variant was the best evidence for the diagnosis of Alport syndrome [8, 10]. Despite the introduction of the standards and guidelines for the interpretation of sequence variants (ACMG/AMP criteria [24]) there is sometimes insufficient data for a conclusion of pathogenicity. The association with haematuria or kidney failure, and a family history of haematuria or renal failure may be the strongest evidence for the diagnosis of Alport syndrome. Microscopic haematuria is found in 95% of those with a pathogenic *COL4A5* variant [8], and at least 67% with a pathogenic heterozygous *COL4A3 or COL4A4* variant [6]. End-stage kidney failure occurs in all males, but many fewer females with a pathogenic *COL4A5* variant [9]. The situation is more complicated in recessive Alport syndrome by the requirement for two *COL4A3 or COL4A4* variants in trans [8]. Renal failure is less common, but recognised increasingly, with pathogenic heterozygous *COL4A3* or *COL4A4* variants [17].

The manual application of the ACMG/AMP guidelines is time-consuming, subject to error and yields inconsistent results [25]. The tools themselves may be flawed [26]. Several web-based tools, such as Alamut (www.interactive-biosoftware.com/alamut-visual/), Varsome (www.varsome.com) [27] and the ClinGen pathogenicity calculator (calculator.clinicalgenome.org) [25], may help in classifying variants by summarising the results for ACMG/AMP criteria such as the computational criteria (Polyphen2, SIFT, Mutation taster, conservation scores, and occurrence in gnomAD and other reference databases) in one site [24]. The ACMG/AMP recognise that “those working in specific disease groups should continue to develop more focused guidance regarding the classification of variants in specific genes“, and thus the Chandos House meeting assessed the ACMG criteria in the context of Alport syndrome and the *COL4A3*- *COL4A5* genes and made the following recommendations (Table 2) including the identification of significant functional domains and mutational hotspots (PM1); the degree of conservation of the protein sequences in different species (PP3); the ‘well-established’ functional assays that indicate pathogenicity (PS3, BS3); whether pathogenic variants are absent from databases with healthy control individuals (PM2) [28] and the threshold in these databases above which variants are likely to be benign (BA1, BS1).

**Table 2:** Standards and guidelines for the interpretation of sequence variants in *COL4A3–COL4A5* in Alport syndrome (modified ACMG/AMP criteria).

PVS1	Null variants (nonsense, frameshift, canonical ±1 or 2 splice site) are pathogenic for Alport syndrome. *These account for more than 20% of the pathogenic variants in COL4A3–COL4A5*.
PS3	Well-established (robust and reproducible) in vitro or in vivo functional studies supportive of a damaging effect on the gene or gene product. *Due to their complexity, functional assays are mostly used for research and not their diagnostic utility. The findings must be specific for the variant being tested and not simply be true for the gene*.
PS4	The prevalence of the variant in affected individuals is increased compared with controls. *Pathogenic variants in the Alport genes are not uncommon in reference databases because of gender-specific (COL4A5) or age-dependent penetrance as well as variable expressivity (such as the lack of haematuria in ~30% of pathogenic COL4A3 and COL4A4 heterozygous variants)*.
PM1	Located in a mutational hotspots or critical and well-established domains without benign variation. *Most Glycine residues in the collagenous domain of the collagen IV α5, α3 and α4 chains should be recognised as critical residues equivalent to a functional domain. The Cysteines in the carboxy NC domain are also critical*.
PM2	Absent from controls (or at extremely low frequency if recessive) in gnomAD, ESP, 1000 Genomes or ExAC. *Monoallelic pathogenic variants in COL4A5 affect at least one in 5000 of the population, and heterozygous pathogenic COL4A3 and COL4A4 variants one in 100, which means some pathogenic variants are present in large reference databases of normals*.
PM5	Novel missense change at an amino acid residue where a different missense change determined to be pathogenic has been seen before. *Glycines can be substituted with 8 other amino acids or with a stop codon. There are many examples of multiple substitutions at the same Glycine residue in the collagenous domain*.
PP2	Missense variant in a gene that has a low rate of benign missense variation and where missense variants are a common mechanism of disease. *The collagen IV α5, α3 and α4 chains are highly conserved from H sapiens (humans) to X tropicalis (frogs), especially the Glycine residues in the collagenous domain and many residues in the carboxy NC domains*
PP4	Patients’ phenotype or family history is highly specific for a disease with a single genetic aetiology. *Applicable in families with history of microscopic haematuria, hearing loss and renal failure. At least 80% of individuals with inherited haematuria can be demonstrated to have a pathogenic variant in one or more of the COL4A3–COL4A5 genes*.
BA1	The allele frequency of a variant is above 5% in normal variant databases. *This threshold is also appropriate for benign variants in the Alport genes. It is not possible to further refine the threshold because of the abundance of hypomorphic variants in all the Alport genes in normal variant databases, and the different modes of inheritance of Alport syndrome*
BP1	Missense variant in a gene for which primarily truncating variants are known to cause disease. *This is not relevant for the Alport genes where both missense and nonsense variants cause disease*.
BP2	Observed in trans with a pathogenic variant for a fully penetrant domain gene/disorder; or observed in cis with a pathogenic variant in any inheritance pattern. *These criteria do not exclude a pathogenic variant in the Alport genes, where variants in cis or trans may be pathogenic and worsen the disease phenotype*.
BP3	In-frame deletions/insertions in a repetitive region without a known function. *This is not relevant for the Alport genes*.
BP5	Variant found in a case with an alternate molecular basis of disease. *Variants in the Alport genes occur commonly in individuals with other inherited renal diseases and at least sometimes worsen disease severity*.

The Chandos House meeting did not revise the weighting system for the ACMG/AMP criteria for Alport variants [24]. Nor did it did it reach consensus on who had the responsibility for requesting a review of Variants of Uncertain Significance for a reclassification to Likely Pathogenic/Pathogenic or Likely Benign/Benign and how often this assessment should be undertaken. Many laboratories considered that it was the clinician’s responsibility to request a VUS review based on the ongoing need for a diagnosis or the availability of new clinical information. On the other hand, clinicians considered that laboratories were more aware of advances in algorithms for defining pathogenicity. Finally, the ACMG/AMP recommendations for reporting incidental findings in Clinical Exome and Genome Sequencing do not include pathogenic variants in the *COL4A3–COL4A5* genes [29] despite these being common and clinically significant.

## Modification of the ACMG/AMP criteria for the *COL4A3–COL4A5* genes and rationale

### Types of pathogenic variants

Missense variants are the commonest changes found in *COL4A3–COL4A5*. For the collagen IV α5 chain, more missense variants are found in the large intermediate collagenous domain (84–88% depending on the database) which is more than expected based on length (*p* ≤ 0.006, LOVD, Table 3) and many of which are Glycine substitutions (95% (278/294) in ARUP, arup.utah.edu/database/). Missense variants in the carboxy non-collagenous domain are also common (11–14%). Missense variants that are not Glycine substitutions are probably the most difficult to classify, but even some Glycine substitutions, especially those adjacent to non-collagenous interruptions, are more difficult. In-silico tools used to assess the effect of a particular amino acid substitution on the protein structure may not take into account the character of the Gly-Xaa-Yaa triplets (a Glycine followed by two other amino acids) typical of the collagen chains and as a result their predictions of the effect on structure may be inaccurate.

**Table 3:** Location of missense variants in the collagen IV α5 chain in different variant databases.

Number of missense variants	Number of amino acids (% of total)	ClinVar	ARUP	HGMD	LOVD
Total	1691 (100%)	487	347	430	639
Amino NC domain	41 (2%)	4 (1%)	0	7 (2%) (*p* = 0.34)	1 (0%) (*p* < 0.0001)
Intermediate collagenous domain	1418 (84%)	427 (88%)	303 (87%)	368 (86%)	565 (88%) (*p* = 0.006)
Carboxy NC domain	231 (14%)	56 (11%)	44 (13%)	55 (13%)	73 (11%) (*p* = 0.17)

*p* values indicate the difference in the number of missense variants found in the region according to this database compared with the expected number based on the number of amino acids in this domain using Fisher’s exact test.

Most nonsense variants in the *COL4A3–COL4A5* genes result in nonsense-mediated mRNA decay and loss of the corresponding collagen IV α chains from affected membranes. Because the α5 chain is part of the α3α4α5 heterotrimer, the α3 and α4 chains with which it normally forms the heterotrimer are imperfectly incorporated or not incorporated at all [30], and there is a compensatory increase in the expression of the α1α1α2 network [31]. This means that males with a *COL4A5* nonsense variant typically have no α3α4α5 (or α5α5α6) network in affected membranes. In females with *COL4A5* heterozygous nonsense variants, however, this loss is segmental depending on lyonisation since clusters of cells produce normal amounts of the α5 chain [32]. In males and females with *COL4A3* or *COL4A4* heterozygous nonsense variants, affected cells result in less of the α3α4α5 network and membrane thinning.

Variants in *COL4A5* have been collated into a number of databases (Table 4), from the literature and submissions from individual laboratories. Currently all databases record variant assessments made by the submitting laboratories. The role of such databases in variant assertions is controversial [33, 34] but they still represent useful repositories. ClinVar is the only database that independently evaluates variants but it has no expert panel assessments for the *COL4A3–COL4A5* variants. Most laboratories also consult their own databases of variants that they have assessed previously.

**Table 4:** Comparison of variant databases for *COL4A5*.

*COL4A5*	ClinVar	ARUP	HGMD	LOVD
Website	www.ncbi.nlm.nih.gov/clinvar/	arup.utah.edu/database/ALPORT/ALPORT_display.php	www.hgmd.cf.ac.uk	www.lovd.nl/COL4A5
Total number of variants	1260	807	905 (1169 for subscribers)	1595
Curated	Submitters’ assessments recorded but a star system depending on number of reports and expert panel assessment	Submitters’ assessments recorded	Yes	Submitters’ assessments recorded
Clinical details available	Includes publications with clinical details	Includes publications	Includes publications	Clinical details from submitters plus publications
Includes benign or likely benign variants	Yes (*n* = 101)	Yes (*n* = 23)	Assessments only available to subscribers	Yes (*n* = 177)
Cost	Freely available online	Freely available online	Publicly-available version up to 3 years previously; otherwise professional version	Freely available online

The databases were accessed in April-May 2020.

### Functional studies

The in vitro or in vivo functional studies used in pathogenicity assessments must be specific for the individual gene variant and not for the gene as a whole. One of the commonest in vitro tests for Alport syndrome is immunohistochemistry of a renal or skin biopsy to demonstrate that the collagen IV α5 chain is absent in X-linked and autosomal recessive Alport syndrome [35, 36]. However this does not confirm that a particular variant is pathogenic but only that the individual has a collagen IV defect consistent with the diagnosis of Alport syndrome.

Among the functional studies that support a variant having a damaging effect on the gene or gene product, in vitro trimerization assays that examine secretion into the extracellular space [37] correlate well with clinical variant severity, especially in distinguishing pathogenic from non-pathogenic Glycine variants in *COL4A5*. In addition, for non-canonical splice sites, minigene assays or sequencing of urinary podocyte or fibroblast mRNA can be used, but are currently only available in specialised laboratories [38]. Increasingly, *COL4A5* variants affecting non-canonical splice sites, up to 11 nucleotides from the intron-exon boundary, are identified in individuals with X-linked Alport syndrome. Sometimes synonymous variants within the coding region result in splicing changes that are also disease-causing. Potential splicing changes can be examined with on-line prediction tools such as MaxEntScan or SpliceSiteFinder, but eventually require confirmation with in vitro assays.

Functional assays used in research include evaluation of abnormalities in electrophoresis and thermal stability of collagen IV (previously common in collagen type I); [39, 40] and mRNA quantitation [41]. Again these assays are not specific for individual gene variants unless a zebrafish, mouse or normal cell line has been engineered to incorporate the variant being assessed.

### Critical and well-established domains and mutational hotspots

**Fig. 1:**
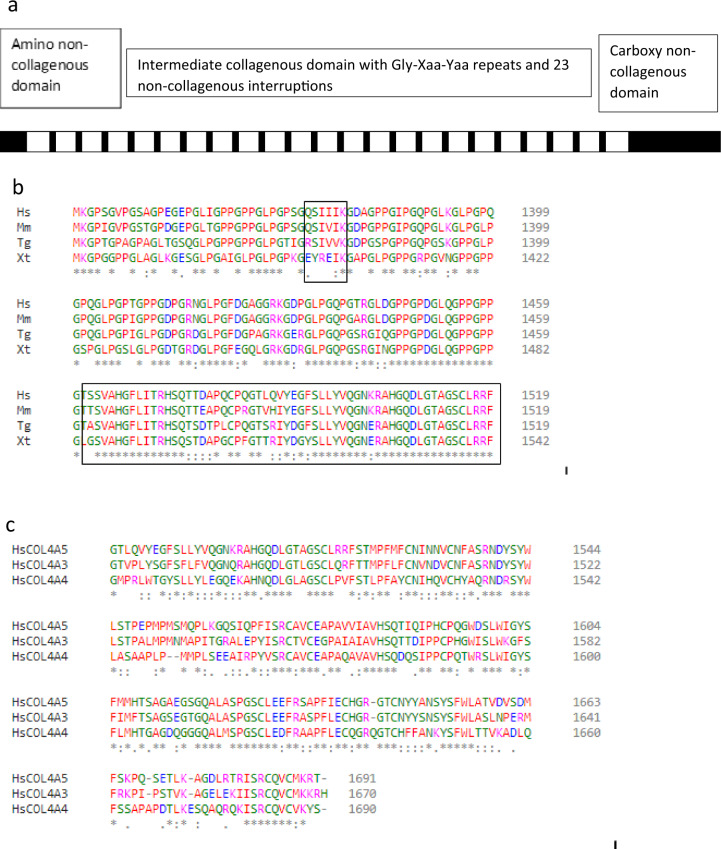
Collagen IV α5 chain. **a** Schematic of collagen IV α5 chain with amino and carboxy non-collagenous domains, and intermediate collagenous sequence with Gly-Xaa-Yaa repeats and 23 non-collagenous interruptions. Gly substitutions adjacent to the interruptions are often hypomorphic with a milder clinical phenotype. **b** Clustal sequence demonstrating that the collagen IV α5 chain is highly conserved between different species both in the collagenous and in the carboxy non-collagenous domains. This sequence also includes a non-collagenous interruption as well as the carboxy terminus (both in boxes). **c** Clustal sequence demonstrating that the human collagen IV α5, α3 and α4 chains are also conserved.

Collagen IV differs from most other collagens in forming a chicken-wire network rather than fibrillar structure. The *COL4A1–COL4A6* genes code for the collagen IV α1–α6 chains. Collagen IV is a heterotrimer with three of the 6 α chains producing a triple helix (α1α1α2, α3α4α5 or α5α5α6) [42].The α3α4α5 network is the main component of the glomerular basement membrane, encoded by the *COL4A3*, *COL4A4* and *COL4A5* genes respectively. The amino acid sequences of these chains are each highly conserved in different species, and also between the individual α5, α3 and α4 chains (Fig. 1, Supplementary Figs. 1 and 2).

Each collagen IV α chain has an amino non-collagenous (NC) domain; an intermediate collagenous domain; and a carboxy NC domain (Fig. 1). The intermediate collagenous domain has the sequence Gly-Xaa-Yaa where X and Y are often Proline or Hydroxyproline. The presence of a Glycine in every third position in the Gly-Xaa-Yaa repeats is critical to triple helix formation because Glycine is the smallest amino acid and fits within the helix interior. The Proline residues contribute flexibility. The three α3, α4 and α5 chains bind together through disulphide bonds at the Cysteine residues and by a sulfilimine bond between Lysine and Methionine residues all within the carboxy NC domain [43]. There are also multiple non-collagenous interruptions in the collagenous domains that confer flexibility and facilitate network formation (Tables 5–7) [44]. Each interruption has adjacent Glycine residues, and some incorporate a Glycine. However the biochemistry is even more complicated because while the transcript for individual α chains is read off forwards 5’ to 3’, from the amino to the carboxy terminus, the three chains bind through disulphide and sulphilimide bonds in the carboxy terminus from which the triple helix winds up ‘backwards’ [45, 46]. Substitution of these critical position 1 Glycine residues within the Gly-Xaa-Yaa sequence distorts and disrupts helix formation. Glycine substitutions in other inherited collagen diseases are typically pathogenic, for example, in *COL1A1* in osteogenesis imperfecta [47]. The effect of Glycine substitutions in positions 2 or 3 or within the interruption is not known. The local environment is also important and Glycine substitutions adjacent to interruptions, where there is more chain flexibility, appear to be less pathogenic [48, 49].

**Table 5:** Non-collagenous domains and interruptions in the collagen IV α5 chain [44].

Non-collagenous interruption (residues)	Residues	Residue number of Gly
Amino -NC domain (1–41)	MKL…DCS	5, 11, 21, 31, 35
I (160–167)	SIIMSSLP	
II (220–223)	LNFQ	
III (243–257)	QISEQKRPIDVEFQK	
IV (282|283)	–	
V (344–355)	LVIPRPGTGITI	350, 352
VI (390–393)	AAVM	
VII (416–419)	ISIP	
VIII (442–453)	PHIPPSDEICEP	
IX (479–487)	DICFNCIGT	486
X (549–554)	DILTFP	
XI (595–599)	GITFK	595
XII (625)	F	
XIII (657–662)	QTITQP	
XIV (706)	I	
XV (753–756)	FALP	
XVI (818)	I	
XVII (853–856)	LDVP	
XVIII (954–960)	PMDPNLL	
XIX (1070–1073)	ISSI	
XX (1189)	F	
XXI (1245–8)	PALE	
XXII (1373–78)	QSIIIK	
Carboxy -NC domain (1461–1691)	TSS…KRT	1467, 1485, 1492, 1500, 1506, 1510, 1513, 1560, 1595, 1602, 1612, 1615, 1617, 1624, 1650, 1642, 1675

This table is from the original report. Since then the Ref Seq has been changed by the addition of 6 amino acids 1264–1264: G>GPTGFQG in the kidney transcript, which alters the numbering.

Interruption IV is formed from two collagenous regions directly adjacent to one another. This results in the Gly-Xaa-Yaa-Gly-Gly-Xaa-Yaa-Gly sequence and a structural kink.

**Table 6:** Non-collagenous domains and interruptions in the collagen IV α3 chain [56].

Interruptions (residues)	Residues	Residue number of Gly
Amino NC (1–42)	MSA…FCD	30, 37
I (160–170)	AKEEDIELDAK	
II (222–223)	VI	
III (245–258)	IVTLTGPDNRTDLK	250
IV (283–287)	YGSEK	284
V (345–353)	EYYDTYQEK	
VI (387–388)	RP	
VII (413–414)	AM	
VIII (442–445)	VFRK	
IX (476–483)	CTQCPYIP	
X (544–547)	LQPE	
XI (587–589)	ALS	
XII (617–618)	GY	617
XIII (649–655)	SVSTPVP	
XIV (698–699)	GI	698
XV (745–749)	AVAMP	
XVI (810–811)	IE	
XVII (848–849)	RS	
XVIII (946–951)	EISHVI	
XIX (1060–1064)	EGTRP	1061
XX (1176–1179)	RAPP	
XXI (1234–1235)	IP	
XXII (1263–1264)	VI	
XXIII (1352–1357)	KIISLP	
Carboxy NC (1439–1670)	ATW…KRH	1445, 1463, 1470, 1478, 1484, 1488, 1491, 1538, 1553, 1573, 1580, 1590, 1593, 1595, 1602, 1618, 1620, 1653

**Table 7:** Non-collagenous domains and interruptions in the collagen IV α4 chain [57].

Interruptions (residues)	Residues	Residue number of Gly
Amino NC (1–61)	MWS…PEK	22, 38, 40, 45, 48, 49
I (176–183)	VFILGAVK	180
II (235–236)	GV	235
III (258–269)	LLVEPPDFCLYK	
IV (294–295)	GI	294
V (359–366)	VTPPLPLK	
VI (400–401)	CA	
VII (429–432)	DSAP	
VIII (457–462)	VIYCSV	
IX (493–496)	ACEP	
X (560–565)	VVSRVK	
XI (605–609)	EDATP	
XII (631–632)	GL	631
XIII (666–673)	ESCNVTYP	
XIV (716–718)	EIP	
XV (740–741)	PV	
XVI (763–764)	AF	
XVII (828–830)	CER	
XVIII (966–971)	AIISQK	
XIX (1014)	R	
XX (1078–1081)	ASHF	
XXI (1196–1197)	DV	
XXII (1222–1223)	PP	
XXIII (1251–1257)	PKDIPDP	
XXIV (1285–1288)	DLLR	
XXV (1370–1379)	ADVDDCPRIP	
XXVI (1404–1405)	GP	1404
Carboxy NC (1460–1690)	PGY…KYS	1461, 1464, 1465, 1483, 1490, 1498, 1508, 1511, 1598, 1606, 1608, 1611, 1612, 1613, 1620, 1636, 1639

Proline substitutions in the collagen IV α5 chain are reported pathogenic much less often than Glycine substitutions (databases.lovd.nl/shared/genes/COL4A5). Proline confers flexibility and its replacement with another amino acid is less problematic than for Glycine. However, Proline residues are also found in both the integrin binding sites and in the GlyProProGlyProPro binding site for glycoprotein VI which is needed for platelet activation [50], but no Proline substitutions have been described in these residues in the *COL4A3–5* genes.

Interestingly, pathogenic variants have been reported for 10 of the 12 Cysteine residues that are critical for cross-linking in the carboxy NC domain (databases.lovd.nl/shared/genes/COL4A5). No substitutions have been described for the Lysine and Methionine residues in the NC domain that form the sulfilimine bond.

Thus, the Chandos House meeting recommended that most Glycine residues in the collagenous domain of the collagen IV α5, α3 and α4 chains should be recognised as critical residues equivalent to a functional domain. However some Glycine substitutions adjacent to NC interruptions represent hypomorphic variants with a milder clinical phenotype [49, 51] such as p.(Gly624Asp) in *COL4A5*. Clinical information is critical for the complete evaluation of variants, and where there are discrepancies, the evidence from many laboratories, such as segregation of haematuria with the variant in multiple families or within many members of the same family, may confirm a pathogenic nature. The 12 Cysteines in the carboxy NC domain should also be considered critical domains, and any substitutions are likely to be pathogenic.

### Hypomorphic variants

Hypomorphic variants are increasingly described in the *COL4A5* gene [51**–**53] although there are, as yet, no definitions or criteria for their identification. They may result in haematuria only, later-onset kidney failure, or GBM thinning rather than lamellation in a male. Variants in the *COL4A3–COL4A5* genes may also modify the pathogenic effects of genes encoding other podocyte or glomerular membrane proteins.

The *COL4A5* hypomorphic variant, NM_000495.5:c.1871G>A substitution (rs104886142); p.(Gly624Asp) results in late-onset kidney failure and is the commonest pathogenic variant causing X-linked Alport syndrome in Central and Eastern Europe [51]. It has previously been considered benign [10] or a VUS. This variant is located adjacent to a non-collagenous interruption in the Gly-X-Y sequence which reduces the effect of the Gly substitution (Table 5). The Chandos House meeting considered that hypomorphic variants, such as p.(Gly624Asp) may still be actionable, requiring renin-angiotensin- aldosterone blockade consistent with published guidelines for other *COL4A5* variants [8, 54].

The nature of other variants such as NM_000495.5:c.2858G>T (rs78972735; p.(Gly953Val)) in *COL4A5* [55], and NM_000092.5:c.1634G>C (rs1800516; p.(Gly545Ala)) and NM_000092.4(COL4A4):c.2996G>A (rs13027659 p.(Gly999Glu)) in *COL4A4* is less clear. All are very abundant in certain ancestries. p.(Gly953Val) is also located immediately adjacent to an interruption in the collagenous domain of the collagen IV α5 chain, but it is not on its own associated with haematuria, kidney failure or GBM lamellation [55], and has conflicting interpretations of pathogenicity in Clin Var (www.ncbi.nlm.nih.gov/clinvar/variation/24573/). Both p.(Gly545Ala) and p.(Gly999Glu) affect Gly residues within the collagenous domain of the collagen IV α4 chain itself. These are considered Benign** (www.ncbi.nlm.nih.gov/clinvar/variation/255015/) and with Conflicting interpretation of pathogenicity* (www.ncbi.nlm.nih.gov/clinvar/variation/191312/) respectively. Thus interpretation of pathogenicity is complicated, and variants while themselves not causing haematuria may still worsen renal failure progression.

## Conclusions

The *COL4A3–COL4A5* genes have particular characteristics that must be considered in assessing variants for a pathogenic or benign nature. Most notable are the importance of the position 1 Glycine substitutions in the collagenous domains, Cysteine substitutions in the carboxy NC domains and the recognition of hypomorphic variants associated with a milder clinical phenotype with microscopic haematuria only, late-onset renal failure or GBM thinning rather than lamellation. Evidence is emerging for the importance of other non-Glycine substitutions especially in the carboxy NC domains but these await further evaluation.

## Supplementary information

Suppl Figure 1

Suppl Figure 2
