# Correction: Consensus statement on standards and guidelines for the molecular diagnostics of Alport syndrome: refining the ACMG criteria

**DOI:** 10.1038/s41431-023-01288-x

**Published:** 2023-01-31

**Authors:** Judy Savige, Helen Storey, Elizabeth Watson, Jens Michael Hertz, Constantinos Deltas, Alessandra Renieri, Francesca Mari, Pascale Hilbert, Pavlina Plevova, Peter Byers, Agne Cerkauskaite, Martin Gregory, Rimante Cerkauskiene, Danica Galesic Ljubanovic, Francesca Becherucci, Carmela Errichiello, Laura Massella, Valeria Aiello, Rachel Lennon, Louise Hopkinson, Ania Koziell, Adrian Lungu, Hansjorg Martin Rothe, Julia Hoefele, Miriam Zacchia, Tamara Nikuseva Martic, Asheeta Gupta, Albertien van Eerde, Susie Gear, Samuela Landini, Viviana Palazzo, Laith al-Rabadi, Kathleen Claes, Anniek Corveleyn, Evelien Van Hoof, Micheel van Geel, Maggie Williams, Emma Ashton, Hendica Belge, Elisabeth Ars, Agnieszka Bierzynska, Concetta Gangemi, Beata S. Lipska-Ziętkiewicz

**Affiliations:** 1 Department of Medicine (MH and NH), The University of Melbourne, Parkville, VIC Australia; 2 Molecular Genetics, Viapath Laboratories, Guy’s Hospital, London, UK; 3 Elizabeth Watson, South West Genomic Laboratory Hub, North Bristol Trust, Bristol, UK; 4 Jens Michael Hertz, Department of Clinical Genetics, Odense University Hospital, Odense, Denmark; 5 Center of Excellence in Biobanking and Biomedical Research and Molecule Medicine Center, University of Cyprus, Nicosia, Cyprus; 6 Medical Genetics, University of Siena, Siena, Italy; 7 Institute de Pathologie et de Genetique ASBL, Departement de Biologie Moleculaire, Gosselies, Belgium; 8 Department of Medical Genetics, and Department of Biomedical Sciences, University Hospital of Ostrava, Ostrava, Czech Republic; 9 Departments of Pathology and Medicine (Medical Genetics), University of Washington, Seattle, WA USA; 10 Institute of Biomedical Sciences, Faculty of Medicine, Vilnius University, Vilnius, Lithuania; 11 Division of Nephrology, Department of Medicine, University of Utah Health, Salt Lake City, UT USA; 12 Clinic of Pediatrics, Institute of Clinical Medicine, Faculty of Medicine, Vilnius University, Vilnius, Lithuania; 13 Department of Pathology, University of Zagreb, School of Medicine, Dubrava University Hospital, Zagreb, Croatia; 14 Nephrology Unit and Meyer Children’s University Hospital, Firenze, Italy; 15 Division of Nephrology and Dialysis, Bambino Gesù Children’s Hospital, IRCCS, Rome, Italy; 16 Department of Experimental Diagnostic and Specialty Medicine (DIMES), Nephrology, Dialysis and Renal Transplant Unit, S. Orsola Hospital, University of Bologna, Bologna, Italy; 17 Wellcome Centre for Cell-Matrix Research, Division of Cell-Matrix Biology and Regenerative Medicine, School of Biological Sciences, Faculty of Biology Medicine and Health, The University of Manchester, Manchester, UK; 18 School of Immunology and Microbial Sciences, Faculty of Life Sciences, King’s College London, London, UK; 19 Fundeni Clinical Institute, Pediatric Nephrology Department, Bucharest, Romania; 20 Centre for Nephrology and Metabolic Disorders, Weisswasser, Germany; 21 Institute of Human Genetics, Technical University of Munich, München, Germany; 22 Nephrology Unit, University of Campania, Naples, Italy; 23 Department of Biology, School of Medicine University of Zagreb, Zagreb, Croatia; 24 Birmingham Children’s Hospital, Birmingham, UK; 25 Departments of Genetics and Center for Molecular Medicine, University Medical Center, Utrecht University, Utrecht, The Netherlands; 26 Alport UK, Gloucester, UK; 27 Medical Genetics Unit, Department of Clinical and Experimental Biomedical Sciences “Mario Serio”, University of Florence, Florence, Italy; 28 Medical Genetics Unit, Meyer Children’s University Hospital, Florence, Italy; 29 Health Sciences Centre, University of UTAH, Salt Lake City, UT USA; 30 Department of Nephrology and Renal Transplantation, University Hospitals Leuven, Leuven, Belgium; 31 Center for Human Genetics, University Hospitals and KU Leuven, Leuven, Belgium; 32 Department of Clinical Genetics, Maastricht University Medical Center, Maastricht, The Netherlands; 33 Bristol Genetics Laboratory Pathology Sciences, Southmead Hospital, Bristol, UK; 34 North East Thames Regional Genetics Laboratory, Great Ormond Street Hospital, London, UK; 35 Department of Physiology, Radboud Institute for Molecular Life Sciences, Radboud University Medical Center, Nijmegen, The Netherlands; 36 Inherited Kidney Disorders, Fundacio Puigvert, Universitat Autonoma de Barcelona, Barcelona, Spain; 37 Bristol Renal Unit, Bristol Medical School, University of Bristol, Bristol, UK; 38 Division of Nephrology and Dialysis, University Hospital of Verona, Verona, Italy; 39 Centre for Rare Diseases, and Clinical Genetics Unit, Medical University of Gdansk, Gdansk, Poland

**Keywords:** Diseases, Alport syndrome

Correction to: *European Journal of Human Genetics* (2021) 29:1186–1197 10.1038/s41431-021-00858-1

The following Acknowledgement was missing:

This research has been supported not financially by “European Reference Network for Rare Kidney Disease, ERKNet”. This ERN is partly co-funded by the European Union within the framework of the Third Health Programme “ERN- 2016 – Framework Partnership Agreement 2017–2021”.

The original article has been corrected.

