# Supplementary figures and images for: Consensus statement on standards and guidelines for the molecular diagnostics of Alport syndrome: refining the ACMG criteria
Source: Eur J Hum Genet. 2021 Apr 15;29(8):1186–97. doi: 10.1038/s41431-021-00858-1 (PMC8384871; doi:10.1038/s41431-021-00858-1)

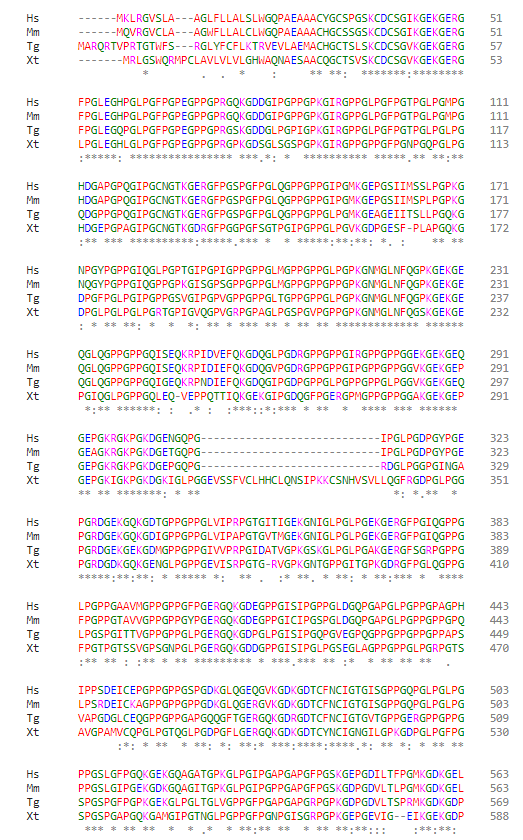


**Amino NC domain**


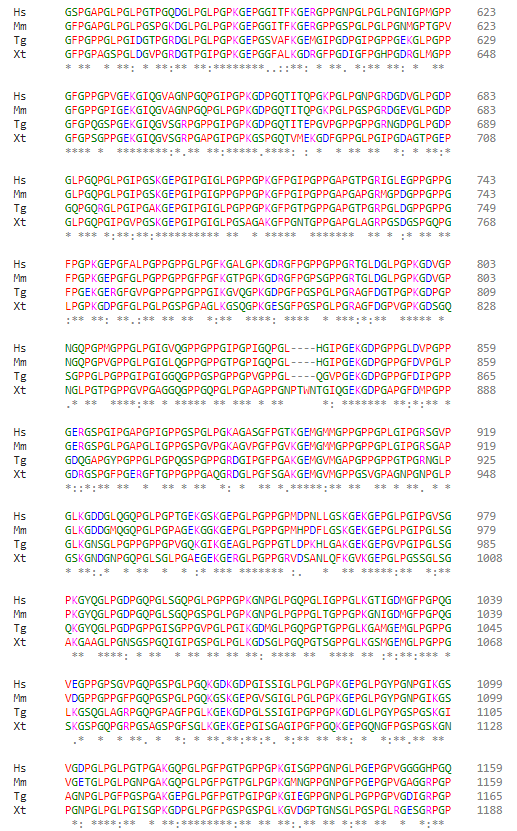

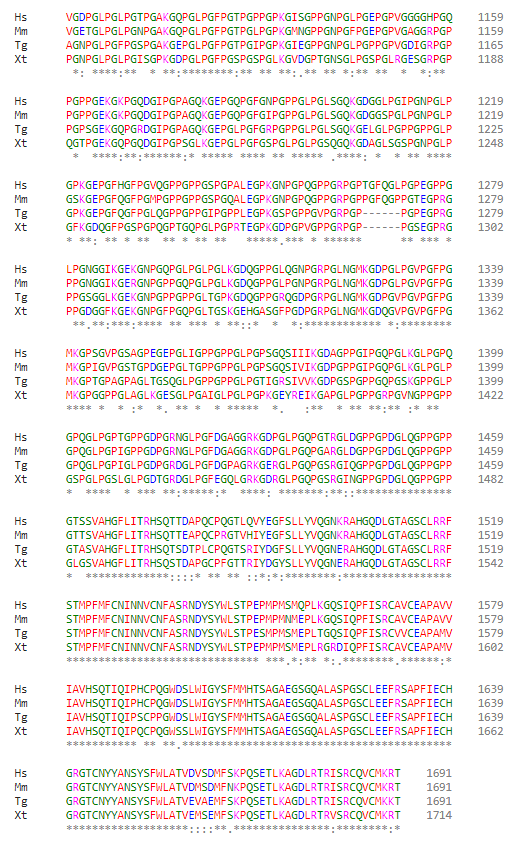


**Carboxy NC domain**

Supplement: Supplementary file 1 — Suppl Figure 1 [file 41431_2021_858_MOESM1_ESM.docx]

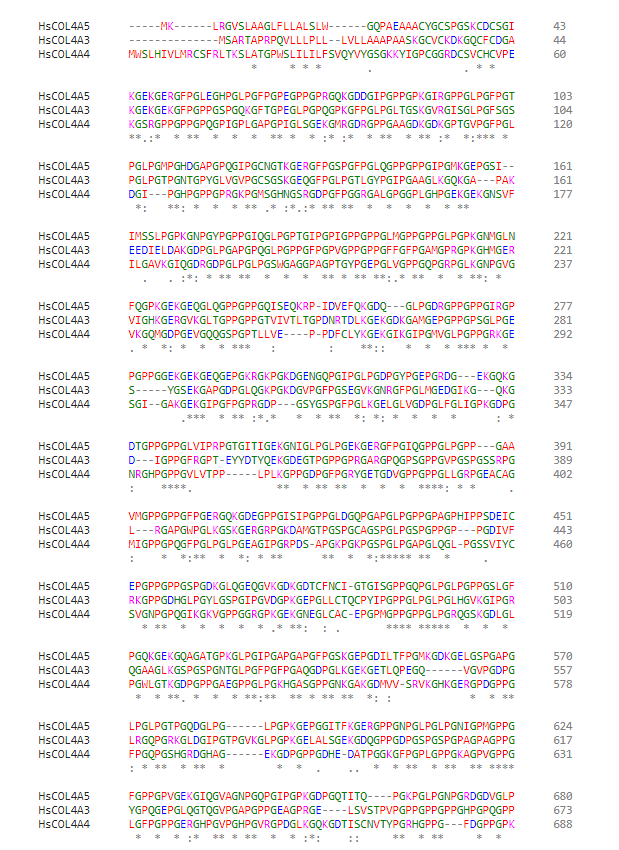


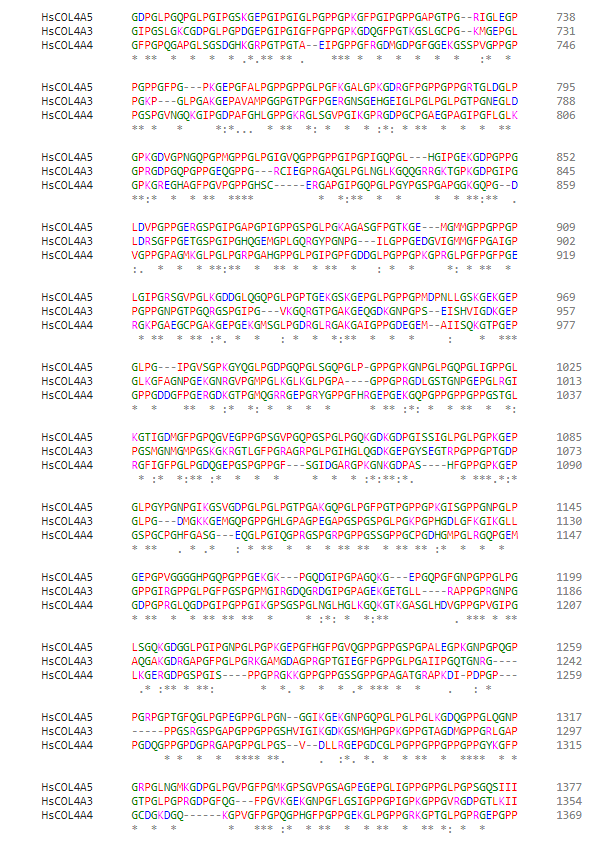


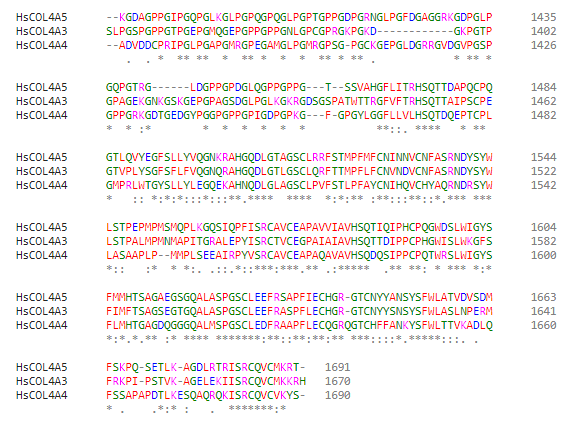

Supplement: Supplementary file 2 — Suppl Figure 2 [file 41431_2021_858_MOESM2_ESM.docx]
